# Metabolite-Mediated Antioxidant-Rich Bacterial Isolates for the Control of Anthracnose Disease and Enhancement of the Post-Harvest Shelf Life of Mango (*Mangifera indica* L.)

**DOI:** 10.3390/plants15071130

**Published:** 2026-04-07

**Authors:** T. Damodaran, Karma Beer, Prasenjit Debnath, Sumit K. Soni, Maneesh Mishra, M. Muthukumar, Nisha Sulakhe, Prabhat Kumar Shukla

**Affiliations:** ICAR—Central Institute for Subtropical Horticulture, Rehmankhera, P.O. Kakori, Lucknow 226101, Uttar Pradesh, India; tdamodaran73@gmail.com (T.D.); dr.biotechsumit@gmail.com (S.K.S.); maneeshmishra.cish@gmail.com (M.M.); muthukumarbt@gmail.com (M.M.); nishasulakhe1997@gmail.com (N.S.); pksmush@gmail.com (P.K.S.)

**Keywords:** mango, *Preistia aryabhattai*, anthracnose, bio-stimulants, shelf life

## Abstract

Mango (*Mangifera indica* L.), being a climacteric fruit, is highly perishable due to rapid ripening and post-harvest diseases like anthracnose, which significantly shorten its shelf life and limit long-distance sea export. To mitigate these constraints, a chemical-free secondary metabolite-based formulation (SMsF) was developed to delay ripening and control post-harvest anthracnose during storage. The SMsF possesses dual-action properties and is derived from the culture filtrate of *Priestia aryabhattai*, exhibiting ACC deaminase activity that restricts ethylene formation. It is also rich in antifungal compounds such as vanillic acid, hydroxybenzoic acid, cryptochlorogenic acid, palmitic acid, and BBIT, which inhibit anthracnose development. Additionally, it contains antioxidants including quercetin, coumaryl quinic acid, oleic acid, and acetylglycitin that enhance shelf life and disease resistance. The efficacy of SMsF was evaluated in mango cv. Banganapalli was stored at 12 ± 1 °C and 85–90% relative humidity under simulated reefer conditions (SRC). Integration of gamma irradiation with SMsF provided superior results in disease control and shelf-life extension. The combined treatment maintained higher fruit firmness (0.86 kg cm^−2^), optimal total soluble solids (14.3 °B), desirable acidity (0.22%), and complete suppression of anthracnose (PDI = 0) up to 40 days of storage under SRC compared with the control. The findings conclusively demonstrate that the synergistic application of SMsF and gamma irradiation effectively regulates ripening, enhances fruit quality, and ensures complete disease suppression, thereby significantly extending storage life. This approach holds strong scientific and commercial significance as a sustainable, residue-free, and export-oriented technology capable of improving long-distance transportation, reducing post-harvest losses, and promoting safe mango trade.

## 1. Introduction

Mango (*Mangifera indica* L.) is an important fruit worldwide, known for its delicious taste and nutritional value [1]. The world produced 54.83 million tons of mangoes in 2020, covering approximately 55 million hectares [2]. According to the Ministry of Agriculture and Farmers Welfare, India remains the world’s largest producer of mangoes, contributing approximately 40–45 percent to global mango production. In the crop year of 2024-25 (second advance estimates), India produced 228.37 lakh tons of mangoes. Mango, being a climacteric fruit, faces substantial post-harvest losses due to a sudden increase in the rate of respiration after harvesting and the incidence of post-harvest diseases [3].

The dangerous post-harvest diseases are anthracnose (*Colletotrichum gloeosporioides*), stem-end rot (*Lasiodiplodia theobromae*), and Alternaria fruit rot (*Alternaria alternata*), which degrade fruit quality during storage and transportation [1]. Among post-harvest diseases, anthracnose caused by *Colletotrichum gloeosporioides* is the most damaging, resulting in economic losses exceeding 40% and significantly impacting mango production worldwide [4]. This pathogen severely compromises fruit quality by causing surface lesions, rot, and premature spoilage, leading to reduced market acceptance in both domestic and international trade [5]. Globally, anthracnose has been reported to cause yield losses of up to 60% in mangoes, particularly during storage and transportation [6].

Agrochemicals (fungicides/pesticides), which are applied predominantly at the post-harvest stage, can create a risk to human health, and at pre-harvest, they pollute the environment due to dipping/drifting or spraying of agrochemicals over the fruits. The challenge of fungicide resistance drives farmers/growers to apply fungicides more frequently at higher concentrations to mitigate losses from fungal diseases [7]. Additionally, a couple of methods have been reported to minimize the post-harvest disease incidence in mango fruits, such as hot-water treatment at 52 ± 1 °C for 10 min, which has been found to be the most convenient and effective method [8]. Post-harvest dipping of harvested Kesar mango fruits with a one-centimeter stalk in Bavistin (1000 ppm) and Dithane M-45 (2000 ppm) solutions was found to be highly effective in controlling anthracnose disease [9]. However, these chemical treatments are not considered safe for human health or the environment, and their residual effects may occur, leading to rejection of the entire consignment during export and resulting in significant economic losses.

To address these issues, research efforts are focused on the use of organic-based, eco-friendly and cost-effective formulations derived from biological control agents (BCAs) that act as sustainable replacements for management of post-harvest diseases in fruits and vegetables [10]. The use of microbial-based antagonists as bio-control agents has emerged as a promising post-harvest approach not only for disease management but also for enhancing shelf life [11]. The antagonist bacteria produce antifungal compounds that prevent the establishment of the pathogen on the host [12]. Currently, few methods are available, such as integrating bacterial antagonists with hot water treatment, which has been shown to provide greater control over post-harvest disease control compared with using individual treatments alone [13]. Naturally occurring antagonists on host surfaces present a promising approach to biological crop protection [14]. A study revealed the antagonistic activity of *Trichoderma harzianum*, *Bacillus subtilis*, and *Pichia anomala*, either individually or in combination, against mango anthracnose, with *Trichoderma harzianum* proving to be the most effective [15]. Additionally, isolate 558 was identified as *Pseudomonas fluorescens*, which significantly reduced anthracnose disease development in mango [16].

As far as ethylene is concerned, it is one of the most important factors that is directly linked to the shelf life of climacteric fruits, especially mango. At lower temperatures and higher humidity, ethylene evolution is reduced. However, little information is available on the role of metabolites in post-harvest ripening of mango. Hydrogen water treatment not only affects the post-harvest ripening characteristics of banana fruit through its reductive properties, but may also influence ethylene synthesis and ethylene action, thereby directly regulating fruit-ripening traits. This study provides a novel strategy for delaying post-harvest ripening of banana fruit during storage [17].

A novel approach of using secondary metabolites from antagonistic bacteria and fungi, the bioactive compounds present in cell-free extracts (CFEs), has been found to effectively inhibit post-harvest pathogens without introducing live microorganisms onto consumable produce [18,19]. Several findings have demonstrated that volatiles produced by bio-control agents, including alcohols, aldehydes, alkenes, esters, phenols, flavonoids, and ketones, tend to increase shelf life and control post-harvest diseases [20,21]. Edible coatings, which are naturally occurring, such as carboxy methyl cellulose (CMC), gum arabic, and chitosan, have also been proven to control post-harvest diseases [22,23]. Additionally, irradiation is an emerging technique that extends shelf life, delays fruit ripening and senescence, and reduces microbial activity as well as insect infestation [24]. The present investigation focused on the identification of bacterial isolates that are rich in antioxidants that have antifungal activity and contain ACC deaminase to regulate ethylene production. This study also envisaged the preparation of secondary metabolite-based formulations using the bacterial isolates and assessment of their ability to control post-harvest disease, i.e., anthracnose incidence and ethylene regulation during storage.

## 2. Results

### 2.1. Bacterial Assessment of 1-Aminocyclopropane-1-Carboxylic Acid Deaminase Activity

**Figure 1:**
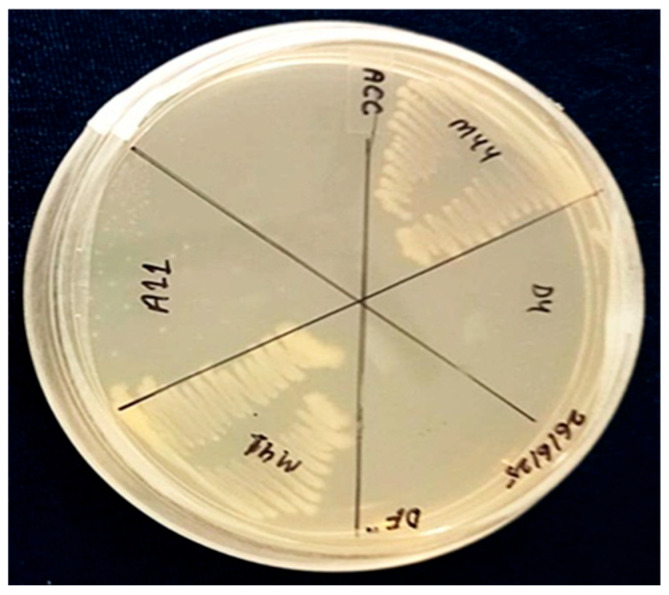
1-aminocyclopropane-1-carboxylic acid deaminase activity of bacterial strains.

In this study, seven rhizobacterial isolates were examined to determine ACC deaminase activity. Remarkably, among the tested isolates, only one (CSR-D44 of *Priestia aryabhattai*) demonstrated proficient growth on DF minimal salt medium augmented with 3 mM ACC as the exclusive nitrogen source, indicative of robust ACC deaminase activity. Subsequently, this singular isolate was selected for subsequent in-depth analysis and investigation (Figure 1).

### 2.2. Assessment of Antagonistic Activity of Bacterial Strains

**Figure 2:**
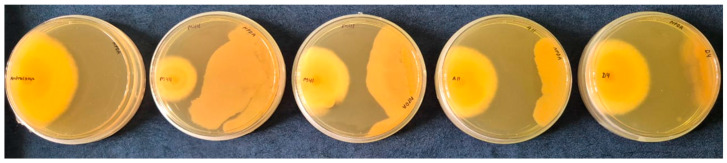
Assessment of antagonistic activity of bacterial strains, viz., CSR-D44, CSR-D41, CSR-A11 and CSR-D4, against phyto-pathogen *C. gloeosporioides*.

The dual-culture technique was used to evaluate the antagonistic potential of bacterial strains, viz., CSR-D44, CSR-D41, CSR-A11, and CSR-D4, against the phytopathogen *C. gloeosporioides*. The radial growth of *C. gloeosporioides* grown alone (control) was compared with the radial growth of *C. gloeosporioides* in the presence of bacterial isolates (treatments) after 5 days of inoculation. The results observed on the culture plate indicated the maximum antagonistic potential in CSR-D44, followed by CSR-D41, CSR-A11 and CSR-D4, respectively (Figure 2). Considering the control value as 100%, 65.79%, 39.47%, 28.94%, and 21.05% reductions in the radial growth of *C. gloeosporioides* were observed in the presence of CSR-D44, CSR-D41, CSR-A11 and CSR-D4, respectively (however, CSR-A11 and CSR-D4 were statistically par to each other (Table 1)).

### 2.3. Inhibitory Effect of the Bacterial Isolates Against Colletotrichum gloeosporioides

**Figure 3:**
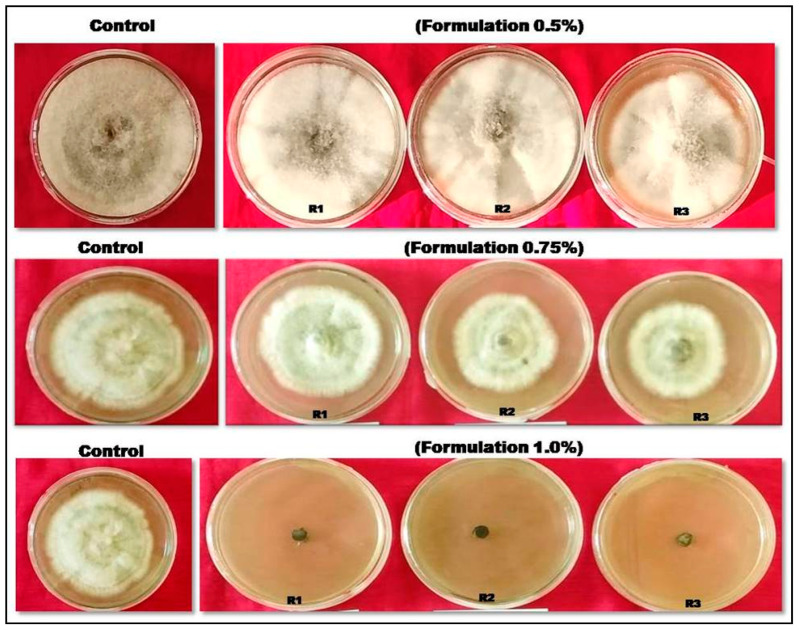
Pictorial representation showing inhibition of mycelial growth of anthracnose in presence of CSR-D44 isolate of *Prestia aryabhattai* cell-free extract (SMsF). In vitro screening revealed that 1.0% concentration of CSR-D44 isolate of *Prestia aryabhattai* cell-free extract completely inhibited the disease.

The inhibitory potential of the bacterial isolates against *C. gloeosporioides* was assessed in vitro with gradual concentrations, viz. 0.5%, 0.75% and 1.0%, of SMsF, following the poison plate technique. The findings indicated that all bacterial isolates exhibited notable efficacy (*p* ≤ 0.05) in restraining the radial expansion of *C. gloeosporioides* on culture plates. The poison plate assay revealed that the secondary metabolite formulation (SMsF) of *Priestia aryabhattai* isolate D44 significantly inhibited the radial growth of *Colletotrichum gloeosporioides*. On the 7th day after inoculation (DAI), fungal growth was progressively restricted as the SMsF concentration increased. At a 1.0% concentration, complete inhibition of fungal growth was observed (Figure 3; Table 1). These results suggest that the SMsF of *Priestia aryabhattai* exhibits strong dose-dependent antifungal activity, with maximum efficacy at 1.0%, highlighting its potential as an effective bio-control agent against anthracnose.

### 2.4. LC-MS-Based Metabolic Profiling of CSR-D44: Chromatographic Analysis and Insights

**Figure 4:**
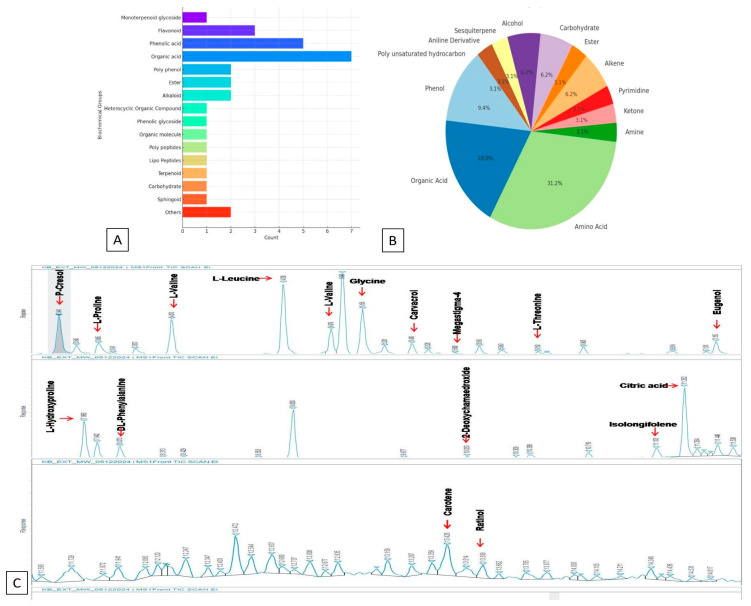
(**A**,**B**) Bar and pie chart illustrating the distribution of identified metabolites in the cell-free extract of *P. arryabati* based on LC-MS analysis; (**C**) GC-MS chromatographic profile illustrating the abundance of various secondary metabolites in crude SMsF.

The LC-MS analysis of the cell-free extract of *Prestia aryabhattaii* identified a diverse range of metabolites, categorized into 16 biochemical groups, including flavonoids, phenolic acids, organic acids, polyphenols, esters, alkaloids, heterocyclic organic compounds, phenolic glycosides, polypeptides, lipoterpenoids, and peptides. A total of 34 bioactive compounds, classified mainly as flavonoids, quinones, phenolic acids, terpenes, antioxidants, and derivatives of lipids and amino acids, were detected (Figure 4A,B; Supplementary Figure S1). These metabolites were identified using the available literature and metabolic databases (mz cloud and mass-bank). Flavonoids were observed as dominant compounds, with phloretin (100%), quercetin diglycoside (53%), and flavonoid quercetin (43%), contributing significantly. Phenolic acids were also abundant, with hydroxybenzoyl acid hydroxide, vanillic acid-o-hexoside, vanillic acid derivative, and coumaryl quinic acid all showing 100% abundance. Organic acids, including palmitic acid (100%), syringic acid (100%), and phloionolic acid (100%), further highlighted the biochemical richness of the extract. Polyphenols, such as caffeoyl-2-hydroxyethane (100%), and alkaloids, including tomatidin (100%) and thebane (100%), contributed significantly. Other identified compounds included peptaibols (poly peptides), iturin A2 (lipopeptides), and daucosterol (terpenoid), which are known for their bioactive properties. The presence of sphingoids (phytosphings (92%)), phenolic glycosides (coumaric acid hexoside-1 (100%), and organic molecules (nuciferin (64%)) further highlights the metabolic diversity of the extract. Overall, the findings suggest that *P. arryabati* contains a wide array of bioactive metabolites with potential pharmaceutical and therapeutic applications. Furthermore, cryptochlorogenic acid, coumaric acid, and hexoside-1 were also detected during the global metabolic imprint search. Furthermore, the bar chart shows the distribution of identified metabolites in the cell-free extract of *P. arryabati*, grouped into 16 biochemical categories. Among these, organic acids account for the highest proportion, at 21.9% of the total metabolites, followed by phenolic acids (18.8%), indicating their major presence. Flavonoids (12.5%) and polyphenols (9.4%) also play significant roles in the extract’s biochemical composition. The presence of flavonoids, polyphenols, alkaloids, and other metabolites suggests potential pharmaceutical and therapeutic applications, emphasizing the bioactive potential of *P. arryabati* (Figure 4A,B).

From the GC–MS chromatogram, (Agilent Technologies, Inc., USA) several metabolites were identified with their corresponding retention time (RT) values. The early eluting compounds included p-cresol (RT ≈ 4.92 min) and L-proline (RT ≈ 5.48 min), followed by L-valine (RT ≈ 8.63 min). A prominent peak corresponding to L-leucine (RT ≈ 10.41 min) was observed, while L-isoleucine (RT ≈ 11.26 min) and glycine (RT ≈ 12.18 min) appeared in the mid-retention region. Other detected metabolites included carvacrol (RT ≈ 13.76 min), megasigma-4 (RT ≈ 14.82 min), L-threonine (RT ≈ 16.05 min) and eugenol (RT ≈ 18.94 min). In the second chromatographic panel, L-hydroxyproline (RT ≈ 5.12 min) and DL-phenylalanine (RT ≈ 6.37 min) were detected, along with 2-deoxy-mannopyranoside derivative (RT ≈ 15.21 min). A strong peak of citric acid (RT ≈ 23.64 min) indicated a comparatively higher abundance, while isolongifolene (RT ≈ 22.48 min) was also observed. In the later region of the chromatogram, caffeine (RT ≈ 19.37 min) and retinol (RT ≈ 20.12 min) were identified. Overall, the chromatographic profile indicated the presence of amino acids, phenolic compounds, organic acids, and bioactive secondary metabolites in the analyzed sample (Figure 4C).

### 2.5. Physical Assessment of Banganapalli Fruits Quality

**Figure 5:**
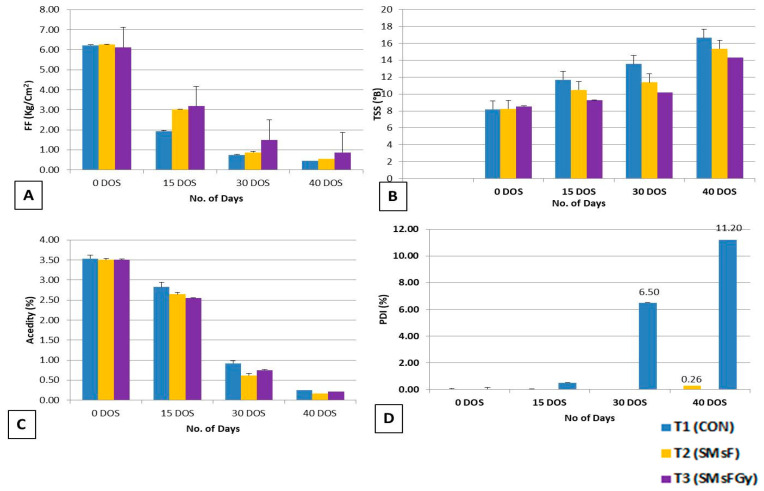
Bar graph showing the dynamic changes in (**A**) total soluble solids (TSSs); (**B**) acidity; (**C**) fruit firmness (FF); and (**D**) Percentage Disease Index (PDI) of mango cv. Banganapalli fruits. Statistical analysis indicated that the values presented in (**A**–**D**) are expressed as the means of five replicates ± standard error, and differences among treatments were significant at *p* ≤ 0.01. At 30 and 40 DOSs, firmness, TSS, acidity, and PDI showed statistically significant variation among treatments.

Generally, low-temperature storage has an impact on fruit firmness (FF) and PLW as the fruits gradually proceed towards ripening. Both parameters play an important role in the shelf life and exportable quality of Banganapalli fruits. In the current investigation, FF did not show any significant changes across all treatments at the time of harvesting. After storage in the reefer container, the fruit firmness (FF) declined over time. The fruit firmness in 15 DOSs was 1.9 kg/cm^2^ in the control, 3.0 kg/cm^2^ in SMsF-treated fruits, and 3.2 kg/cm^2^ in the combined treatment (SMsFGy). A similar trend was recorded in 40 DOSs, wherein the CON, the SMsF treatment alone, and the SMsFGy treatment showed reduced firmness values of 0.45 kg/cm^2^, 0.54 kg/cm^2^, and 0.86 kg/cm^2^, respectively (Figure 5A).

### 2.6. Biochemical Assessment of Banganapalli Fruit Quality Parameters During Storage in Reefer Container

**Figure 6:**
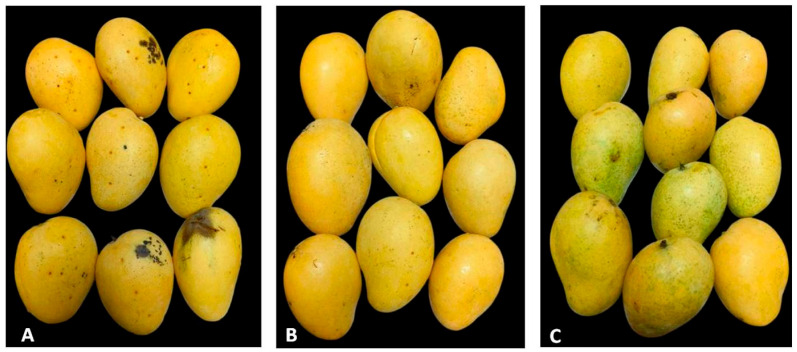
Post-harvest condition of Banganapalli mango fruits after 40 days of storage (DOSs) in a reefer container: (**A**) T_1_ (CON)—untreated control fruits after 40 DOSs; (**B**) T_2_ (SMsF)—fruits treated with secondary metabolites formulation (SMsF); (**C**) T_3_ (SMsFGy)—fruits treated with secondary metabolites formulation and gamma irradiation.

During storage under simulated reefer conditions, total soluble solids (TSSs) showed a progressive increase across all treatments (Figure 5B). At 15 DOSs, CON recorded 11.69 °B, whereas SMsF and SMsFGy exhibited lower values of 10.48 °B and 9.25 °B, respectively. By 40 DOSs, TSSs further increased, reaching 16.6 °B in CON, followed by 15.3 °B in SMsF and 14.3 °B in SMsFGy. In contrast, total acidity (TA) showed a declining trend throughout the storage period (Figure 5C). At 15 DOSs, TA was highest in CON (2.8%), followed by SMsF (2.6%) and SMsFGy (2.5%). After 40 DOSs, acidity levels decreased markedly, with CON fruits recording 0.25%, compared with 0.17% in SMsF and 0.22% in SMsFGy. However, it has already been reported that ethylene plays a crucial role in mitigating abiotic and biotic stresses. The current study modulated ethylene production in stored mango at 13 °C and 85% RH in a reefer container. The SMsF treatment, along with an ethylene absorbent and gamma radiation, delayed the ripening process in mango cv. Banganapalli up to 40 DOSs. SMsF retained the firmness of the treated fruits compared with CON fruits, whereas SMsFGy-treated fruits were found to be better in retaining fruit FF (0.86 kg/cm^2^), TSSs (14.3 °B), acidity (0.22%) and PDI (0-nil) compared with CON fruit samples’ FF (0.45 kg/cm^2^), TSS (16.6 °B), acidity (2.8%) and PDI (11.2%) at 40 DOSs (Figure 6A–C).

### 2.7. Assessment of Post-Harvest Disease (PHD) Incidence in Banganapalli Fruits in Reefer Container

Up to 15 DOSs in the reefer container (temperature: 13 °C; RH: 90%), the SMsF and SMsFGy treatments showed no symptoms of anthracnose, while CON exhibited a slight incidence of 0.5%. By 30 DOSs, the disease had progressed further in CON (6.5%), whereas SMsF and SMsFGy remained disease-free. Conversely, at 40 DOSs, CON recorded the highest disease incidence at 11.2%, followed by SMsF at 0.26%, while SMsFGy (0%) remained completely disease-free throughout the investigation (Figure 5D). This confirmed that SMsF, along with gamma ray irradiation, provides protection against pathogens causing diseases. The result showed highly significant variability in the disease incidence during the observation period. Statistical analysis of the data revealed a significant impact of SMsF on disease incidence (*p* < 0.05), with a tendency for increased disease incidence as storage time prolonged. The post-harvest condition of Banganapalli mango fruits after 40 days of storage in a reefer container showed clear differences among the treatments. Fruits in A (T_1_—control) exhibited noticeable deterioration with visible dark spots and decay symptoms, indicating a higher post-harvest disease incidence and reduced quality. In contrast, fruits in B (T_2_—SMsF) treated with the secondary metabolites formulation maintained a comparatively better appearance with minimal surface damage and improved overall fruit quality. The best preservation was observed in C (T_3_—SMsFGy), where fruits treated with the secondary metabolites formulation combined with gamma irradiation retained good color, firmness, and showed the least disease symptoms, suggesting that the combined treatment was most effective in extending shelf life and reducing post-harvest deterioration after 40 days of storage (Figure 6A–C).

### 2.8. Impact of SMsF Treatment on Phenolic Compounds in Banganapalli Fruits

The HPLC analysis revealed significant variations in the phenolic composition across different sample types. The results indicate that the SMsFGy-Peel sample exhibited the highest concentrations of key phenolic compounds, suggesting that the SMsFGy treatment was the most effective in enhancing phenolic retention. Notably, ellagic acid was significantly elevated in SMsFGy-Peel (1877.41 µg/g), which was nearly double the amount found in SMsF-Peel (1012.23 µg/g) and significantly higher than in CON-Peel (192.05 µg/g). Similarly, chlorogenic acid showed a substantial increase in SMsFGy-Peel (521.99 µg/g) compared with SMsF-Peel (29.77 µg/g) and CON-Peel (737.65 µg/g). This suggests that the SMsFGy treatment enhanced the retention of this key phenolic compound. Additionally, gallic acid and caffeic acid concentrations remained relatively high in SMsFGy-Peel (225.31 µg/g and 104.74 µg/g, respectively), further supporting the effectiveness of SMsFGy in preserving these bioactive compounds. However, a notable decline in catechin acid was observed in SMsFGy-Peel (52.53 µg/g) compared with SMsF-Peel (204.49 µg/g), suggesting a compound-specific impact of the treatment. *p*-coumaric acid was present in SMsFGy-Peel (13.51 µg/g) but absent in pulp samples, indicating its preferential retention in the peel. Overall, the findings highlight that the SMsFGy treatment was the most effective in enhancing phenolic content, particularly for ellagic acid, chlorogenic acid, and gallic acid (Table 2). The significant variation in chlorogenic acid further reflects treatment-mediated metabolic modulation. The highest chlorogenic acid content was recorded in CON (737.65 μg/g), likely due to advanced ripening and elevated oxidative stress stimulating the phenylpropanoid pathway. In contrast, SMsF-treated fruits showed a markedly lower level (29.77 μg/g), suggesting delayed ripening and reduced stress conditions. Interestingly, SMsFGy recorded an intermediate but comparatively high value (521.99 μg/g), possibly due to mild irradiation-induced stimulation of phenolic biosynthesis. Collectively, these findings indicate that SMsF treatments modulate the phenylpropanoid pathway by selectively enhancing key defense-related phenolics, such as ellagic acid, while suppressing others during delayed ripening and storage (Table 2).

The phenolic profiling revealed that ellagic acid exhibited the strongest positive correlation with the SMsF treatments in both peel and pulp. In mango peel, ellagic acid increased markedly from 192.05 μg/g in CON to 1012.23 μg/g in SMsF and further to 1877.41 μg/g in SMsFGy, while in pulp, it rose from 56.54 μg/g (CON) to 151.92 μg/g (SMsF) and 172.70 μg/g (SMsFGy). This substantial enhancement suggests its pivotal role in strengthening antioxidant defense and contributing to improved storage stability, particularly under the combined SMsF and gamma irradiation treatment. In pulp, gallic acid and catechin acid also showed a positive association, especially in SMsFGy, whereas gallic acid (in peel), caffeic acid, and epi-catechin acid generally exhibited reduced levels compared with CON, indicating a negative correlation with the SMsF treatments. In pulp, gallic acid and catechin acid also showed a positive association, particularly under the SMsFGy treatment. Conversely, gallic acid (in peel), caffeic acid, and epi-catechin acid generally exhibited a negative correlation with the SMsF treatments, showing reduced concentrations compared with CON (Table 2).

### 2.9. GC-MS Profiling of Crude SMsF: Metabolite Identification and Analysis

The untargeted profiling of crude SMsF using GC-MS analysis revealed a diverse range of metabolites classified into 13 biochemical groups (Supplementary Table S2). A total of thirty-three (33) volatile organic compounds (VOCs) from different chemical groups were detected. Amino acids constituted a major fraction, with L-proline, 2TMS derivative (8.32%), L-leucine, 2TMS derivative (7.41%), and glycine, 3TMS derivative (4.73%), contributing significantly. Organic acids were also abundant, with citric acid, 4TMS derivative (5.52%), being the most prominent. Among phenols, p-cresol, TMS (4.34%), was the dominant compound. Other notable metabolites included phenylalanine, 2TMS derivative (4.23%), hexanoic acid, 3-trimethylsilyloxy, trimethylsilyl ester (0.8%), and isolongifolene, 4,5,9,10-dehydro- (0.92%), indicating the presence of alkenes, ketones, esters, carbohydrates, and sesquiterpenes. The presence of 9-cis-retinol (0.72%) and retinol acetate (0.77%) suggests potential bioactive properties. Overall, the analysis highlights the complex metabolic profile of SMsF, with amino acids, organic acids, and phenols being the dominant contributors. Among the identified compounds, carvacrol (1.04), L-hydroxy proline (2.94), and phenylalanine (1.3) might be responsible for the antimicrobial properties of SMsF. Furthermore, the presence of abundant essential amino acids and other organic acids like L-proline (8.43), valine (3.43), leucine (7.41), glycine (4.73), L-threonine (0.2), citric acid (5.52), 2-amino-succinic acid 4-ethyl ester (0.88), etc., were encountered in the chromatographic profile of SMsF (Figure 4C; Supplementary Table S2).

## 3. Discussion

Anthracnose, caused by the fungus *Colletotrichum gloeosporioides*, is a significant post-harvest disease that poses a serious challenge to the marketing and storage of mangoes. This fungal pathogen can lead to substantial economic losses by affecting the quality and shelf life of mango fruits. Lately, there has been an increasing curiosity in developing alternative and sustainable methods to manage anthracnose in mangoes, with a focus on bio-control agents such as *Bacillus* species. In the current study, four *Bacillus* isolates were used for an antagonistic study against *C. gloeosporioides*. *Bacillus* sp. are known to inhibit the growth of phyto-pathogens such as *Ascochyta blight*, *Xanthomonas campestris*, and *Fusarium* sp. [25,26,27,28]. Additionally, *Bacillus* strains have also been found to produce bioactive molecules like lipo-peptides that restrict the growth of phyto-pathogens.

*P. aryabhattai* is recognized for producing bioactive secondary metabolites with antimicrobial properties against a variety of phyto-pathogens, such as *Verticillium* species and *Macrophomina phaseolina* [29,30]. Studies have emphasized the importance of assessing different concentrations of cell-free secondary metabolites to ascertain their inhibitory effects on phyto-pathogens. Various studies have shown concentration-dependent inhibition of phyto-pathogens, indicating that higher concentrations of secondary metabolites can result in increased growth inhibition of fungi [31].

The current study demonstrated that among all Bacillus isolates, *P. aryabhattai* exhibited the highest potential to inhibit the radial growth of *C. gloeosporioides* under in vitro conditions using the dual-culture method. Furthermore, the antagonistic activity of varying concentrations of cell-free secondary metabolites (SMsF) from *P. aryabhattai* was found to significantly inhibit the growth of *C. gloeosporioides*. Chromatographic profiling of the cell-free extract of *P. aryabhattai* revealed the presence of multiple bioactive metabolites, including flavonoids, phenolic acids, organic acids, polyphenols, and alkaloids, which may contribute to its antifungal properties. Notably, flavonoids such as phloretin, quercetin diglycoside, and flavonoid quercetin were identified, along with phenolic acids like hydroxybenzoic acid hydroxide, vanillic acid-o-hexoside, vanillic acid derivative, and coumaryl quinic acid. The presence of organic acids, including palmitic acid, syringic acid, and phloionolic acid, further emphasized the biochemical diversity of the extract. Additionally, polyphenols such as caffeoyl-2-hydroxyethane and alkaloids, including tomatidin and thebane, were detected, suggesting the potential antifungal and antioxidant activity. Several of these identified compounds, including phloretin, hydroxybenzoic acid hydroxide, vanillic acid derivative, BBIT, peptabols, palmitic acid, and syringic acid, have been previously reported for their antifungal properties [32] (Supplementary Table S1). Moreover, defensive metabolites such as coumaryl quinic acid, coumaric acid hexoside-1, tomatidin, olic acid, and β-linoleic acid, which play crucial roles in plant defense against biotic and abiotic stresses, were identified [33]. Additionally, antioxidant molecules, including paeoniflorin, flavonoid quercetin, quercetin diglycoside, acetylglycitin, naringenin, syringic acid, and daucosterol, were also observed. Compounds such as cryptochlorogenic acid, coumaric acid, and hexoside-1 were also identified in the current study, which are known to participate in lignin biosynthesis [34], and we assumed that these compounds may play a crucial role in restricting anthracnose disease and retaining fruit firmness during prolonged storage.

Furthermore, GC-MS profiling of the crude SMsF identified various VOCs with antifungal properties, along with phenolic compounds, flavonoids, and an enrichment of amino acids. These components could be the likely contributors to the antifungal activity of SMsF and its role in extending the shelf life of fruits. Specifically, phenolic compounds such as p-cresol, carvacrol, eugenol, and epicatechin were detected. Notably, antifungal compounds, including carvacrol, L-hydroxy proline, and phenylalanine, were identified, which might be responsible for the antimicrobial properties of SMsF. Additionally, the presence of essential amino acids and organic acids, such as L-proline, valine, leucine, glycine, L-threonine, citric acid, and 2-amino-succinic acid 4-ethyl ester, was observed in the chromatographic profile of SMsF, further highlighting its biochemical complexity and potential biological activities. Overall, these findings suggest the extensive bioactive potential of *P. aryabhattai* and its secondary metabolites, suggesting promising applications in the development of antifungal agents and plant protection strategies. Compounds like carvacrol, a plant metabolite having a broad-spectrum antimicrobial effect [35], and a few amino acids (L-proline, L-valine, L-leucine, L-isoleucine, L-proline, glycine, phenyl alanine and L-hydroxy proline) were also detected (Figure 4C and Supplementary Table S2). However, exogenous application of proline inhibits enzymatic browning in cut potatoes during cold storage [36]. These bioactive compounds may play a crucial role in the long-term storage of mango by reducing post-harvest anthracnose disease and extending shelf life. This finding aligns with previous research, where production of metabolites such as 2-propanone and 2-methylpiridine, which act as volatile organic compounds (VOCs), restricted the growth of the fungus and induced host resistance [37]. An earlier report suggests that the *B. aryabhattai* TCI-16 strain has the capability to produce chitin deacetylase, an enzyme responsible for breaking down chitin, a key component in fungal cell walls. Furthermore, another strain like *B. aryabhattai* AYG1023.2 prevents the development of blue mold caused by *Penicillium expansum* by producing antifungal VOCs like 2-nonanol [38]. Several studies have demonstrated the efficiency of microbial VOCs in controlling post-harvest diseases in fruits, and all VOCs produced by bio-control agents can be categorized as alcohols, aldehydes, alkenes, esters, phenols, flavonoids, ketones, etc. [39,40]. The bio-formulation of selective metabolites from *P. aryabhattai* has demonstrated efficacy in increasing the shelf life of fruits and vegetables by controlling anthracnose disease caused by fungal pathogens [41]. By utilizing these bio-formulations on susceptible crops, farmers can effectively manage post-harvest diseases, reduce spoilage, and enhance marketable yield and food security. However, by leveraging the ACC-deaminase activity of *P. aryabhattai*, it may be possible to develop strategies for managing post-harvest diseases effectively, potentially through the modulation of ethylene levels and other stress responses in plants. The application of *P. aryabhattai* with ACC-deaminase activity could offer a sustainable approach to enhance crop quality and reduce post-harvest losses due to diseases. Moreover, bio-control agents derived from *P. aryabhattai* offer a sustainable and environmentally friendly approach to disease management in agriculture. These bio-formulations have the potential to replace conventional chemical pesticides, reducing reliance on synthetic chemicals and minimizing the environmental impact of agricultural practices [42]. Beneficial bacteria with 1-amino-cyclopropane-1-carboxylic acid (ACC) activity, a precursor of ethylene, are a preferred choice for regulating ethylene production during post-harvest fruit storage. Ethylene enzymatic activity leads to the production of α-ketobutyrate and ammonia by reducing ACC levels [43]. Here, in the current study, similar activity was also found for CSR-D44 *Priestia aryabhattai* (ON399221), having the ACC deaminase property, which was assumed to regulate ethylene production (Supplementary Figure S1), and the formulation prepared from the cell-free extract of *P. aryabhattai* enriched with several antifungal, antioxidant, and lipopolypeptide molecules helped to effectively restrict post-harvest anthracnose disease development and extended the shelf life of mangoes.

Ethylene is present in various plant parts, including stems, leaves, flowers, seeds, and fruits, where it accelerates fruit ripening [44]. Its levels also increase in response to biotic and abiotic stresses [45]. Climacteric fruits exhibit a rapid surge in ethylene production during the climacteric phase, causing them to ripen quickly and have a limited shelf life. Therefore, controlling ethylene levels is essential for enhancing fruit stability and prolonging shelf life. Earlier, it was reported that down-regulation of ACC synthase [46] and ACC oxidase [47] could restrict ethylene production. But in the present investigation, the bacterial enzyme ACC deaminase was utilized to modulate plant ethylene levels [48]. Similarly, activity in the *P. aryabhattai* strains was also observed in the current study (Supplementary Figure S1). The bio-formulation prepared from the selected metabolites from *P. aryabhattai* demonstrated efficacy in increasing the shelf life of fruits (mango) and restricting post-harvest disease development. Furthermore, the developed formulation was subjected to chromatographic profiling to elucidate the story behind the activity in the expansion of Banganapalli′s shelf life and, also, to restrict PHD. LC-MS profiling revealed the presence of a few antifungal/antimicrobial compounds like phloretin [49], hydroxy benzoic acid hydroxide (massbank), vanillic acid derivative and palmitic acid [50], and syringic acid [51] (Supplementary Table S2). These antifungal compounds might create a stressful environment for the pathogen to grow during long-term storage in a reefer container. Along with the antifungal compounds, some SMs recognized for their antioxidant properties, like flavonoid quercetin [52], quercetin diglycoside [52], and acetylglycitin and daucosterol (terpenoid) [53] were also encountered in the *P. aryabhattai* extracts, which could provide favorable conditions during post-harvest storage of Banganapalli fruits by acting as antioxidant molecules to reduce oxidative senescence during storage. Furthermore, a few SMs were also detected, which could act in defense or inner-immunity boost-up, like oleic acid [54], β-linoleic acid, etc. Furthermore, cryptochlorogenic acid [34] was also detected, with a past report of lignin biosynthesis. The current investigation revealed SMsF enriched with SMs used in the post-harvest treatments of mango cv. Banganapalli extended shelf life and restricted post-harvest diseases up to 40 DOSs compared with the CON fruit samples. This is the first record of the highest ever shelf-life enhancement, which offers scope for long-distance marketing. SMsF (Copyright: 2614/2024-C0/L) is a metabolite-based organic wash formulation, rich in secondary metabolites, aiming to delay early ripening and manage post-harvest diseases (anthracnose) as a one-step solution to prolong the shelf life of Mango var. Banganapalli. The formulation enriched with the metabolic extract of *B. aryabhattai*, which has high ACC deaminase activity, helped to reduce ethylene production [55]. The SM-enriched bio-formulation prepared from the culture filtrate of *P. aryabatti,* with the polypeptides present in the SMs, enabled the formation of the ACC deaminase enzyme, which catalyzes the conversion of ACC to ammonia and α-ketobutaric acid, thereby downregulating ethylene production by converting the precursor molecule (Supplementary Figure S1).

In the peel surface of the treated mangoes (T3—SMsFGy), several protective metabolites were enhanced, including β-D-glucopyranosiduronic acid (0.06), D-(–)-erythrofuranose, tris(trimethylsilyl) ether (isomer) (0.09), 3-(3,5-difluorophenyl)-L-alanine, 2TMS (0.10), 1,2,3-heptanetriol, 3TMS (0.08), and D-(–)-tagatofuranose, pentakis(trimethylsilyl) ether (isomer) (0.12). In contrast, the combined treatment (T3 + γ-irradiation at 400 Gy) led to the expression of a distinct set of metabolites, namely, α-D-(–)-lyxopyranose, 4TMS derivative (0.08), 8-hydroxy-3-(4-hydroxypentyl)isochroman-1-one, 2TMS (0.08), ribonic acid, 2,3,4,5-tetrakis-O-(trimethylsilyl) (0.06), talose, 5TMS derivative (1.73), and β-D-glucopyranoside, phenylmethyl 2,3,4,6-tetrakis-O-(trimethylsilyl) (0.07). These bioactive compounds were uniquely expressed in the treated samples and were absent in the control mango peel. This clear shift in metabolite profiles highlights the treatment-induced enhancement of metabolite richness at the mango peel surface, which is likely associated with the observed extension of fruit shelf life.

It also helped maintain a slower TSS spike than in CON fruits during prolonged storage. The current investigation results are in line with the study by [56], which found that the application of bio-formulation reduced anthracnose infestation and enhanced the post-harvest quality of avocado fruits. Similar results have been reported earlier, where hot water treatment with CFB packaging elevated the shelf life for up to 5 days; the use of an edible coating using xanthan gum and pomegranate peel extract extended the shelf life of mango cv. Safeda up to 15 DOSs at 22 °C; food irradiation (400 Gy) extended the shelf life of mango [57]; an edible coating (25% honey been coating) extended the shelf life of mango [58]; and the shelf life of mango was also increased by using neem oil [59]. Earlier reports revealed that shelf life was extended and PHD was reduced to a certain extent, depending on the cultivar, up to no more than 22 days. However, the present investigation demonstrated that mango cv. Banganapalli could be stored successfully for up to 40 days under simulated reefer conditions with the integrated SMsF and gamma irradiation treatment. While previous studies have reported improvements in mango storage and disease management [57,58,59], the extended storage achieved under the current treatment conditions highlights its effectiveness and practical relevance for long-distance transportation and export.

## 4. Materials and Methods

### 4.1. Isolation and Characterization of Plant Growth-Promoting Rhizobacteria

*Colletotrichum gloeosporioides* was sourced from the Division of Crop Protection, ICAR-Central Institute for Sub-tropical Horticulture, Rehmankhera, Lucknow, Uttar Pradesh. The bacterial antagonists CSR-A11 *Lysinibacillus fusiformis* (KU745624), CSR-A16 *Lysinibacillus sphaericus* (KU745625), CSR-M16 *Bacillus licheniformis* (KC768636), CSR-D41 *Bacillus megaterium* (ON399222), CSRD-44 *Priestia aryabhattai* (ON399221), CSR-D43 *Priestia flexa* (ON399223), and CSR-D4 *Bacillus licheniformis* (MK9553781) used in this study were previously isolated from the rhizospheric soil of salt-tolerant cultivars and suppressive soils of banana fusarium wilt [12]. The isolates were obtained from the culture collection of the microbiology laboratory of the ICAR-Central Soil Salinity Research Institute, the Regional Research Station, Lucknow, for further analysis. The cultures were systematically maintained on nutrient agar for subsequent use. Additionally, fungal spores were cultured from 10-day-old potato dextrose agar (PDA) cultures that were incubated at 28 ± 2 °C.

### 4.2. In Vitro Assay on the Bacterial Isolates Against Colletotrichum gloeosporioides

The antagonistic potentials of rhizospheric isolates CSR-A11, CSR-A16, CSR-M16, CSR-D41, CSR-D44, CSR-D43 and CSR-D4 against *C. gloeosporioides* were assessed under in vitro conditions using the dual-culture technique, as outlined by Dhingra and Sinclair [20], with slight modifications. In this method, a mycelial disc measuring three millimeters in diameter from *C. gloeosporioides*, obtained from a 7-day-old culture, was inoculated on one end of potato dextrose agar (PDA-Hi-Media) medium (20 mL) in a 90 mm diameter plate. Simultaneously, the bacterial isolates were streaked on the opposite end of the plates separately. The pathogen and antagonist were both inoculated equidistantly from the periphery of the Petri plate. Control plates were inoculated solely with the phyto-pathogen. The inoculated plates were incubated at 28 ± 2 °C for 7 days. Data were collected to calculate the percent inhibition (PI) of radial growth using Formula (1):

(1) $$PI=\frac{\left(R1-R2\right)}{R1}\times 100$$

where R1 represents the radial growth of the pathogen in the control, and *R*2 represents the radial growth of the pathogen in dual-culture experiments with antagonists.

### 4.3. Preparation of Cell-Free Culture Filtrates and Determination of Inhibitory Concentration of Colletotrichum gloeosporioides

The bacterial isolate with high antagonistic ability, identified by the dual-plating method, was cultivated on nutrient agar media at 30 °C for 48 h to propagate active bacterial colonies. These colonies were introduced into the nutrient broth medium and incubated at 30 °C at 110 rpm for 48 h to establish an inoculum broth. After incubation, the culture filtrates were obtained by centrifuging the nutrient broth culture media at 6000 rpm for 10 min at 4 °C [60]. Subsequently, the supernatant was filtered through 0.4 µm pore size membrane filters, resulting in the cell-free culture broth [61]. The resulting filtrate was then stored as a stock solution in the refrigerator.

The poison food technique was employed to assess the inhibitory effect of the cell-free culture filtrate of CSR-D44, the identified isolate, against *C. gloeosporioides*. Different concentrations (0.5%, 1%, 1.5%, 2%, 2.5%, and 3%) of the filtrate were incorporated into sterilized molten potato dextrose agar (PDA), and the mixture was poured into sterile Petri dishes. After solidification, 5 mm blocks of *C. gloeosporioides* were placed at the center of each plate. The plates were sealed and incubated at 28 ± 2 °C for 7 days. The growth inhibition was quantified using a formula, comparing the pathogen’s growth in treated and control media using the specified Formula (1) mentioned above.

### 4.4. Qualitative Assessment of 1-Aminocyclopropane-1-Carboxylic Acid Eaminase Activity

The qualitative assessment of ACC deaminase activity involves evaluating a microorganism’s ability to utilize ACC as its sole nitrogen source, indicating the presence of the ACC deaminase enzyme. The distinct bacteria were screened for ACC deaminase activity on the sterile minimal DF (Dworkin and Foster) salts media (DF salts) per liter, comprising 4.0 g of KH_2_PO_4_, 6.0 g of Na_2_HPO_4_, 0.2 g of MgSO_4_·7H_2_O, 2.0 g of glucose, 2.0 g of gluconic acid and 2.0 g of citric acid with trace elements, including 1 mg of FeSO_4_·7H_2_O, 10 mg of H_3_BO_3_, 11.19 mg of MnSO_4_·H_2_O, 124.6 mg of ZnSO_4_·7H_2_O, 78.22 mg of CuSO_4_·5H_2_O, and 10 mg of MoO_3_ (pH 7.2), amended with 3 mM ACC as the sole nitrogen source [62]. This medium, after sterilization, was poured into Petri dishes and allowed to solidify before the microorganism was streaked onto the surface. The size and intensity of the halos formed around colonies were indicative of relative enzyme activity, facilitating comparison among different isolates. The inclusion of positive and negative controls ensured the reliability of the results. This straightforward qualitative approach offers an effective means of evaluating ACC deaminase activity in microorganisms.

### 4.5. Preparation of Bio-Stimulant and Treatment Protocol in Mango Fruits

The secondary metabolites obtained from the bacterial isolate exhibiting high antagonistic potential against anthracnose disease and high ACC deaminase activity were used to develop a bio-stimulant for post-harvest treatment of mango fruit for experimentation. The selected bacterial isolate was grown in nutrient broth media and incubated for 24 h. Furthermore, it was formulated using adjuvants and polymers, in accordance with the patent-protected protocol (Patent No. 202411046233 dt. 14 June 2024).

### 4.6. Irradiation Treatment

As per the APEDA, New Delhi-approved packhouse facility equipped with irradiation infrastructure, dosimeters were placed by a qualified dosimetrist at designated reference positions within the mango boxes for each product lot. This ensured that the mangoes received a minimum absorbed dose of 400 gray of irradiation, in compliance with Rule 7 CFR Parts 305 and 519, as published in the Federal Register, Vol. 72, No. 47, pp. 10902–10903.

### 4.7. Fruit Materials and Post-Harvest Treatments

A batch of mature green mango (*M. indica*, cv Banganapalli) attains physiological maturity approximately 100–110 days after fruit set, when the shoulders become well developed and the fruit reaches around 10°Brix of total soluble solids. The mangoes were procured from the mango orchards located at Medipalle, the Ranga Reddy district of the Telangana region of India (GPS co-ordinates: 17°22′18.6″ N and 78°11′35.4″ E), where good agricultural practices (GAPs) as per the protocol developed by ICAR-Central Institute for Subtropical Horticulture, Lucknow, were implemented to produce exportable quality mango fruits. Fruits were harvested using the mango harvester to avoid mechanical injury to the peel, and de-sapping was performed to drain sap. The hot water treatment at 53 °C for 3 min was performed, and after that, the fruits were treated with 1% SMsF for 10 min, followed by drying. After packing the fruits in five-ply CFB boxes, the fruits were subjected to gamma irradiation at a dose of 400 gray of irradiation (as per the compliance with Rule 7 CFR Parts 305 and 519, as published in the Federal Register, Vol. 72, No. 47, pp. 10902–10903) at the mango pack house owned by Innova Agri. Bio. Park, Malur Road, Karnataka, India, approved by APEDA- New Delhi. The packed boxes were stacked and loaded into the reefer container, with the relative humidity (RH) at 90% and the temperature at 12.0 ± 1 °C.

### 4.8. Treatment Details

The fruits were dipped in SMsF (1%) for 10 min; contrarily, the fruits treated with clean tap water for 10 min were considered as the control in the current study. Non-KMnO_4_-based ethylene absorbents were used in all treatments except the control. Each treatment was replicated five times (each replication having three fruits).

T1: Control [CON];

T2: Secondary metabolite-based formulation (1%) [SMsF];

T3: Secondary metabolite-based formulation (1%) + gamma irradiation (400 Gy) [SMsFGy].

### 4.9. Effect of SMsF on Physical and Chemical Parameters of Mango

Quality parameter analysis involved the systematic evaluation of morphological aspects, total soluble solids (TSSs) [63], titratable acidity (TA) [64], ascorbic acid content (AC) [63], and fruit firmness (FA) using appropriate techniques [65]. Moreover, a thorough evaluation of the shelf life of Mango cv. Banganapalli was conducted by continuously observing the changes in appearance and texture, recording any signs of decay, mold, or alterations, and assessing any physiological weight loss during storage in both control and treated fruits.

### 4.10. Qualitative Assessment of Anthracnose Disease

Screening samples for anthracnose symptoms was done using percent disease incidence scores. The percentage disease index (PDI) was calculated for anthracnose by adopting the following formula devised by Mckinney et al. [66] (Table 3).

$$Percentage\;disease\;index\;(PDI)=\frac{Sum\;of\;all\;numerical\;ratings}{Number\;of\;samples\;observed\times maximum\;disease\;grade}\times 100$$

### 4.11. Extraction and Chromatographic (LC/MS) Profiling of Secondary Metabolites

The bacterial isolate efficient in inhibiting *C. gloeosporioides* growth was selected for further LC/MS analysis. The samples were prepared according to the guidelines outlined by the Sophisticated Analytical Instrumentation Facility (SAIF) at the ICAR-Central Institute for Subtropical Horticulture (CISH), Lucknow, India. The extraction of bacterial secondary metabolites for subsequent LC-MS profiling involved a multi-step protocol to ensure the efficient recovery and identification of these compounds. Initially, bacterial isolates were introduced into 100 mL of LB broth and incubated at 28 ± 2 °C for 5 to 7 days in a shaking incubator set at 110 rpm. Subsequently, the cells were separated from the supernatant containing the secondary metabolites through centrifugation at 3900 rpm for 20 min at 4 °C. The resulting supernatants were adjusted to pH 2 by adding 1 N HCl. To precipitate secondary metabolites, the supernatants were incubated overnight at 4 °C. The resulting acidified supernatants underwent two extraction steps using an equal volume of ethyl acetate.

The organic phase obtained from the liquid–liquid extraction was carefully collected, and a minute portion of the solvent-free extract was transferred into micro-centrifuge tubes for subsequent detailed analysis. The remaining extracted residue could be stored at 4 °C for later use in antimicrobial assays. The electrospray ionization liquid chromatography–mass spectrometry (ESI-LC–MS) analysis of the thin-layer chromatography (TLC)-purified sample underwent analysis using a MICROMASS QUATTRO II triple quadrupole mass spectrometer (Micromass, Beverly, MA, USA), coupled with a JASCO PU-980 HPLC pump JASCO (Japan), at the Sophisticated Analytical Instrumentation Facility, the ICAR-Central Institute for Subtropical Horticulture (CISH), Lucknow, India. The chromatographic separation employed a WATER SPHERISORBODS 2 column (250 × 4.6 mm, 5 μm), Waters Corporation (Milford, USA) using an acetonitrile:water + 0.1% formic acid solvent system. A gradient elution approach was adopted with a flow rate maintained at 1.0 mL/min. Monitoring was conducted using a photodiode array within the wavelength range of 200–650 nm, with specific recording at 220 nm. Mass spectra were acquired across the 80–1000 Da range within a scanning time of 2.5 s. The ESI capillary was adjusted to 3.5 kV, and the cone voltage was set at 40 V.

### 4.12. Gas Chromatography–Mass Spectrometry Profiling of Volatile Organic Compounds (VOCs)

TMS derivatives of the sample extracts were prepared. Approximately 5 mg of the sample was suspended in 40 µL of the solution of methoxylamine hydrochloride in pyridine (20 mg/mL). The mixture was shaken for 2 h at 37 °C before adding 70 µL of 2,2,2-trifluoro-N-methyl-N-trimethylsilylacetamide (MSFTA) for 30 min. The resulting derivatized mixture of metabolites was subjected to analysis on GC-MS, and data analysis was performed using standard procedures. Volatile organic compounds (VOCs) were identified by comparing their mass spectra with the NIST library database, and only compounds with a matching score of ≥80% were considered reliably identified.

### 4.13. Characterization of Phenolic Compounds in Banganapalli Mango Peel and Pulp Under Different Treatments

To quantify phenolic compounds, high-performance liquid chromatography (HPLC) was employed, utilizing external standards. The phenolic compounds were analyzed using a Shimadzu HPLC system (Model SCL 10 AVP, Japan), equipped with a Rheodyne manual injector and photodiode array (PDA) detector. Reference standards of various phenolic compounds like gallic acid, chlorogenic acid, catechin, epicatechin, caffeic acid, ellagic acid and p-coumaric acid, along with L-ascorbic acid, were purchased from Sigma-Aldrich, India Branch, Mumbai. HPLC-grade solvents (methanol and water) were procured locally. Stock solutions of 1000 μg mL^−1^ were prepared by dissolving 25 mg of each reference standard in 25 mL of HPLC-grade methanol. Working solutions of 5, 10 and 50 μg mL^−1^ for each reference standard (phenolic compounds) were prepared by subsequent dilution in methanol. Chromatography was performed at a flow rate of 1.0 mL/min and a wavelength of 280 nm, utilizing a linear gradient of the mobile phase consisting of a 0.01 M potassium dihydrogen orthophosphate solution (A) and acetonitrile–water (75:25, *v*/*v*) (B) in an 80:20 ratio. The area beneath each peak was utilized for a quantitative study, and the retention times of the standards were used to identify various components in the extracts, which can be calculated by the given Formula (2).

(2) $$Concentration\;(mg/100\;g)=\frac{Area\;of\;sample\times concentration\;of\;standard\times dilutions}{Area\;of\;sample\times volume\;made\times weight\;of\;sample}\times 100$$

### 4.14. Statistical Analysis

For the in vitro (pathogen) and in vivo fruit experiments, a Completely Randomized Design (CRD) was followed. Data from both in vitro and in vivo experiments (fruit physical and biochemical parameters) were analyzed using analysis of variance (ANOVA) to evaluate the efficiency of bacterial isolates against phyto-pathogens. Treatment means were compared using Tukey’s HSD test. All statistical analyses were performed using the SPSS software-20.

## 5. Conclusions

The present study demonstrated the effectiveness of a secondary metabolite-based formulation (SMsF) derived from *Priestia aryabhattai* for improving post-harvest quality and controlling anthracnose in mango (*Mangifera indica* L.) cv. Banganapalli. The formulation exhibited dual functionality by regulating fruit ripening and suppressing disease development due to the presence of multiple bioactive metabolites with antifungal and antioxidant properties. The ACC deaminase activity of the formulation contributed to the regulation of ethylene biosynthesis, thereby delaying the ripening process and maintaining fruit quality during storage. Under simulated reefer conditions (12 ± 1 °C and 85–90% RH), the integration of SMsF with gamma irradiation effectively preserved key quality attributes, such as fruit firmness, total soluble solids, and titratable acidity, while completely suppressing anthracnose incidence. The combined treatment enabled the storage of mango fruits for up to 40 days, demonstrating its strong potential for maintaining quality during long-term storage.

Overall, the results highlight that metabolite-based bio-smart formulations integrated with physical preservation technologies can serve as an eco-friendly and residue-free alternative to conventional chemical treatments for post-harvest disease management. This approach offers significant advantages for reducing post-harvest losses and supporting long-distance transportation of mango fruits, particularly for export through sea shipments. Furthermore, the technology holds promising prospects for strengthening the mango export chain by ensuring better fruit quality and extended shelf life. Future studies should focus on large-scale validation under commercial storage and transportation conditions and explore the applicability of similar metabolite-based formulations for other climacteric fruits to develop sustainable post-harvest management strategies.

## 6. Future Prospects

Future research should focus on molecular validation of induced resistance mechanisms, particularly antioxidant pathways and ethylene regulation, through ACC quantification and *acdS* gene expression analysis. Advanced metabolomic and transcriptomic studies are needed to identify key bioactive compounds responsible for disease suppression. Different mango cultivars and seasons will help confirm consistency and commercial applicability. Integration with other sustainable post-harvest technologies may further enhance export quality shelf-life management.

## Figures and Tables

**Table 1:** Percent inhibition of *Colletotrichum gloeosporioides* in presence of bacterial strains.

S. No.	Treatment	Radial Growth of *C. gloeosporioides* After 5 Days (in mm)	Percent Inhibition (PI) of Radial Growth of *C. gloeosporioides*
1.	*C. gloeosporioides* (control)	3.633 ± 0.088 a ^#^	-
2.	CSR-D44	1.600 ± 0.208 d	65.79
3.	CSR-D41	2.500 ± 0.1 c	39.47
4.	CSR-A11	2.800 ± 0.058 bc	28.94
5.	CSR-D4	3.033 ± 0.145 b	21.05

^#^ Value shown in each column, followed by different letters, is significantly different at *p* ≤ 0.01 and the column value is the mean of three replicates ± Std. error.

**Table 2:** Status of phenolic compounds in Banganapalli mango treated by SMsF.

	Treatment Details	Gallic Acid	Chlorogenic Acid	Catechin Acid	Epi-Catechin Acid	Caffeic Acid	Ellagic Acid	p-Coumaric Acid
Mango peel	T1 (CON)	307.39 ± 1.47 a ^#^	737.65 ± 0.85 a	128.30 ± 0.58 b	451.16 ± 1.93 a	189.63 ± 0.48 a	192.05 ± 1.42 c	49.37 ± 1.08 b
	T2 (SMsF)	181.79 ± 3.58 c	29.77 ± 0.91 c	204.49 ± 0.64 a	376.35 ±1.67 c	114.59 ±1.22 b	1012.23 ±2.95 b	74.28 ± 0.64 a
	T3 (SMsFGy)	225.31 ± 3.56 b	521.99 ± 1.28 b	52.53 ± 0.61 c	428.22 ± 1.24 b	104.74 ± 1.25 c	1877.41 ± 2.15 a	13.51 ± 0.59 c
Mango pulp	T1 (CON)	27.47 ± 0.69 c	1.08 ± 0.11 b	8.39 ± 0.55 b	5.38 ± 0.69 a	3.37 ± 0.15 a	56.54 ± 1.38 c	ND
	T2 (SMsF)	40.31 ± 0.47 b	2.07 ± 0.16 a	5.29 ± 0.61 b	2.13 ± 0.40 c	0.45 ± 0.07 b	151.92 ± 1.36 b	ND
	T3 (SMsFGy)	67.35 ± 0.52 a	0.80 ± 0.01 c	24.53 ± 0.45 a	4.17 ± 0.14 b	3.07 ± 0.25 a	172.70 ± 1.23 a	ND

^#^ Values shown in each column, followed by different letters, are significantly different at *p* ≤ 0.01, and the column values are the means of five replicates ± Std. error. ND: Not Detected.

**Table 3:** Disease rating scale for mango anthracnose.

Grade	0	1	2	3	4	5
Percentage of infection on fruits	No infection	Up to 5	6–10	11–20	21–50	>50

## Data Availability

Data are contained within the article and Supplementary Materials.

## Supplementary Materials

The following supporting information can be downloaded at https://www.mdpi.com/article/10.3390/plants15071130/s1: Figure S1: Mechanism of delay in ripening by regulation of hormones (ethylene and IAA) by CSR-D44 *Priestia aryabhattai* (ON399221); Table S1: Identified bioactive compounds, peak values, retention time (RT), and intensity in the cell-free extract of *P. aryabhattai* along with their corresponding activities; Table S2: Untargeted metabolic profiling of crude SMsF using GC-MS analysis, categorized by biochemical groups.

## Abbreviations

HWT	Hot water treatment
DAIs	Days after inoculation
DOSs	Days of storage
ACC Deaminase	1-aminoicyclopropane-1-carboxylate deaminase
TSSs	Total soluble solids
FSSAI	Food Safety Standard Authority of India
FF	Fruit firmness
GAPs	Good agricultural practices
FAO	Food and Agriculture Organization
PDA	Potato dextrose agar
HPLC	High-performance liquid chromatography
LCMS	Liquid chromatography–mass spectroscopy
SMsF	Secondary metabolites-based formulation
