# Supplementary material for: Metabolite-Mediated Antioxidant-Rich Bacterial Isolates for the Control of Anthracnose Disease and Enhancement of the Post-Harvest Shelf Life of Mango (Mangifera indica L.)
Source: Plants (Basel). 2026 Apr 7;15(7):1130. doi: 10.3390/plants15071130 (PMC13074554; doi:10.3390/plants15071130)
Supplement: Supplementary file 1 [file plants-15-01130-s001.zip › Supplementary Table.pdf]

Supplementary Table S1: Identified bioactive compounds, peak values, retention time (RT), and intensity in the cell-free extract of *P. aryabhattai* along with their corresponding activities.

**Treatments/ Isolates**	**Tentative compounds identified**	**Peak value**	**RT (min)**	**Intensity (%)**	**Activity**	**Reference**
**CSRD-44**	Paeoniflorin	479.8	15.3	60	Terpenes, antioxidant, immunoregulation	Kong et al. 2020; Wen et al. 2019<br>Zhang and Wei 2020, Mass bank
	Phloretin	274.8	17.785	100	Phenolic compound, phloretin inhibited the growth, anti-fungal anti biofilm formation, anti-bacterial	Liu et al., 2021
	Flavonoid quercetin	303.9	20.16	43	Antioxidant	Scigelova et al., 2016<br>Singh et al., 2021
	Cryptochlorogenic acid	353.8	32.31	97	Lignin biosynthesis	Drapal et al., 2019
	Tomatidin	415.8	19.27	100	Glyco-alkaloid (Plant defense)	Mass Bank
	Hydroxy benzoic acid hydroxide	300.9	21.51	100	Antimicrobial Activity	Mass Bank
	Vanillic acid-o-hexoside	330.9	25.06	100	4HBA also has various biological properties including hypoglycemic, anti-inflammatory, antiviral and antioxidative activities	Meeprathom et al., 2018
	Vanillic acid derivative	329.7	27.67	100	Vanillic acid (VA) is a flavoring agent found in edible plants and fruits. Anti-bacterial activity of VA against several pathogen microorganisms.	Mass Bank, Chen et al., 2021
	Coumaryl quinic acid	338.9	29.35	100	Specialized plant metabolites, In plants, CQAs play a defensive role against biotic or abiotic stress	Alcazar Magana et al., 2021<br>Uleberg et al., 2012.
	Quercetin diglycoside	329.8	15.55	53	Flavonoids; antioxidant; Quercetin is a powerful antioxidant, so it potently provides plant tolerance against several biotic and abiotic stresses	Singh et al., 2021
	p-Cresol	107.0	3.50	80	Phenolic compound	Korytowska et al., 2019
	2-butyl-1-benzisothiazolin-3-one (BBIT)	207.7	19.27	100	BBIT offers antimicrobial activity against fungi, yeasts and bacteria.	Mass Bank
	Thebane	311.9	19.62	100	Phenolic compound	Mz cloud;<br>Tak et al., 2019
	Coumaric acid hexoside-1	325.7	23.20	100	Polyphenol<br>*p*-Coumaric acid is one of the phenolic compounds formed during	Osterc et al., 2008<br>Trobec et al., 2005

					the defense strategy of the plant against wounding, lignin biosynthesis	
	Caffeoyl-2-htdroxyethane	339.9	30.25	100	Phenolic compound	Mass Bank
	3-o-(4’-0-caffeoyl glucosyl) quinic acid	341.0	23.66	57	Cyclitol, a cyclic polyol	Mass Bank
	Acetylglycitin	488.8	30.25	43	Antioxidant	Mass Bank
	2’-3/-6-Trimethoxyflavone	312.6	32.31	65	Flavone	Mass Bank
	Nuciferin	295.6	36.51	64	Alkaloid	Mass Bank
	Enniatin A	687.5	31.81	12	Secondary metabolites of *Fusarium sp.*	Sorensen and Giese, 2013
	Epi-Catechin	289.0	14.6	65	Flavonoids	Mass bank
	Crytptochlorogenic acid	353.0	28.51	100	Lignin biosynthesis	Drapal et al., 2019
	Peptaibols	1438.4	17.47	22	Antifungal	Mukherjee and Kenerley, 2010
	Iturin A2	1043.6	29.01	6	Iturin A is an antifungal lipopeptide biosurfactant https://doi.org/10.1016/B978-0-12-822921-7.00008-8	Mass Bank
	Myrifralignan-A	390.4	36.51	22	Secondary metabolites	Marulasiddaswamy et al., 2021
	Parakmerin-A (Lignan)	279.6	16.79	50	Secondary metabolites	Mass Bank
	Naringenin	272.8	36.51	63	It induced reactive oxygen species (ROS) accumulation and Salicylic acid mediated plant pathogen resistence	Sun et al., 2022
	Syringic acid	199.2	11.75	100	Phenolic Compound; synthesized via shikimic acid pathway in plants. Antioxidant and antimicrobial activity	Srinivasulu et al., 2018
	Daucosterol (Terpenoid)	541.9	17.22	10	Natural Sterol and an antioxidant molecule fight against free redicals	Omari et al., 2022
	Stearicdonic acid methyl ester	291.2	8.66	15	Stearidonic Acid methyl ester is a rare polyunsaturated fatty acid of the ω-3 series	Mass Bank
	Isomaltose	341.0	23.66	56	Disaccharide, source of energy	Mass bank
	Oleic acid	281.7	19.60	7	Induced constitutive Defense signaling	Kachroo et al., 2008
	B-Linoleic acid	279.6	16.79	50	Linolenic acid is directly involved in biotic stress responses by the fact that it is a direct substrate for jasmonate synthesis.	Jennifer et al., 2017
	Palmitic acid	255.6	16.79	100	Anti-microbial property	Ma et al., 2016

					https://www.ncbi.nlm.nih.gov/pmc/articles/PMC8176049/	
	Phloionolic	313.8	31.78	100	Plant metabolite	Mass bank
	Phytosphings	316.1	11.75	92	Secondary metabolite	Mass bank

N. B: Metabolites were tentatively identified using spectral comparisons with mzCloud and MassBank mass Spectral Library. The table presents the targeted metabolites along with their corresponding *m/z* values, retention times (RT), and relative peak intensities (%). A value of 100% denotes the base peak intensity within each chromatogram, and all other metabolites are expressed as relative percentages normalized to this base peak (base peak normalization). Compound annotations were assigned based on matching mass spectral data and retention characteristics with available spectral libraries and previously published reports.

Supplementary Table S2: Untargeted metabolic profiling of crude SMsF using GC-MS analysis, categorized by biochemical groups.

Tentative Compounds	Biochemical Group	R.T.	Area %
p-Cresol, TMS	Phenol	3.441	4.34
Carvacrol, TMS derivative		5.436	1.04
Eugenol TMS		7.155	1.39
Epicatechin. TMS		19.7	1.54
Sulfuric acid, 2TMS	Organic Acid	3.54	1.03
(2E)-3-Cyclohexyl-2-butenoic acid, TMS		5.281	1.11
2,6,6-Trimethylcyclohex-1-ene-1-carboxylic acid, TMS		5.943	0.51
Hexanoic acid, 3-trimethylsilyloxy, trimethylsilyl ester Organic caid		6.405	0.8
2,6,6-Trimethylcyclohex-1-ene-1-carboxylic acid, TMS acid		5.943	0.51
Citric acid, 4TMS derivative		11.262	5.52
L-Proline, TMS derivative	Amino Acid	3.665	1.43
L-Valine, 2TMS derivative		4.078	3.43
L-Leucine, 2TMS derivative		4.708	7.41
L-Isoleucine, 2TMS derivative		4.979	2.4
L-Proline, 2TMS derivative		5.043	8.32
Glycine, 3TMS derivative		5.156	4.73
L-Hydroxyproline, (E)-, 3TMS derivative		7.868	2.94
DL-Phenylalanine, TMS derivative		8.07	1.3
L-Threonine, 3TMS derivative		6.15	0.2
Phenylalanine, 2TMS derivative		9.05	4.23
N-(4-Aminobutyl)hydroxylamine	Amine	3.747	0.21
Ethanone, 1-(3-methylphenyl)	Ketone	3.873	0.72
Uracil, 2TMS derivative	Pyrimidine	5.528	0.4
Megastigma-4,6(E),8(Z)-triene	Alkene	5.68	0.22
1,3a-Ethano-3aH-indene, 1,2,3,6,7,7a-hexahydro-2,2,4,7a-tet		7.101	0.26
2-Amino-succinic acid 4-ethyl ester, 2TMS	Ester	5.816	0.88
β-D-Galactopyranoside, methyl 2,6-bis-O-(trimethylsilyl)	Carbohydrate	6.914	0.51
2-Deoxychamaedroxide		10.033	0.26
9-cis-Retinol	Alcohol	11.538	0.72
Retinol, acetate		13.589	0.77
Isolongifolene, 4,5,9,10-dehydro- (Sesquiterpene)	Sesquiterpene	11.103	0.92
N-(3-Aminopropyl)-4-methyl-2-nitroaniline, 2TMS (Aniline derivative)	Aniline Derivative	8.313	0.2
Carotene, 1,1',2,2'-tetrahydro-1,1'-dimethoxy	Poly-unsaturated hydrocarbon	13.428	2.38

N.B: Specific metabolites identified from NIST library with >600 SI & PSI indicating fair enough in identification of metabolites which are targeted in this table with % area of metabolites identified at a specific retention time.
