# Supplementary figures and images for: Metabolite-Mediated Antioxidant-Rich Bacterial Isolates for the Control of Anthracnose Disease and Enhancement of the Post-Harvest Shelf Life of Mango (Mangifera indica L.)
Source: Plants (Basel). 2026 Apr 7;15(7):1130. doi: 10.3390/plants15071130 (PMC13074554; doi:10.3390/plants15071130)

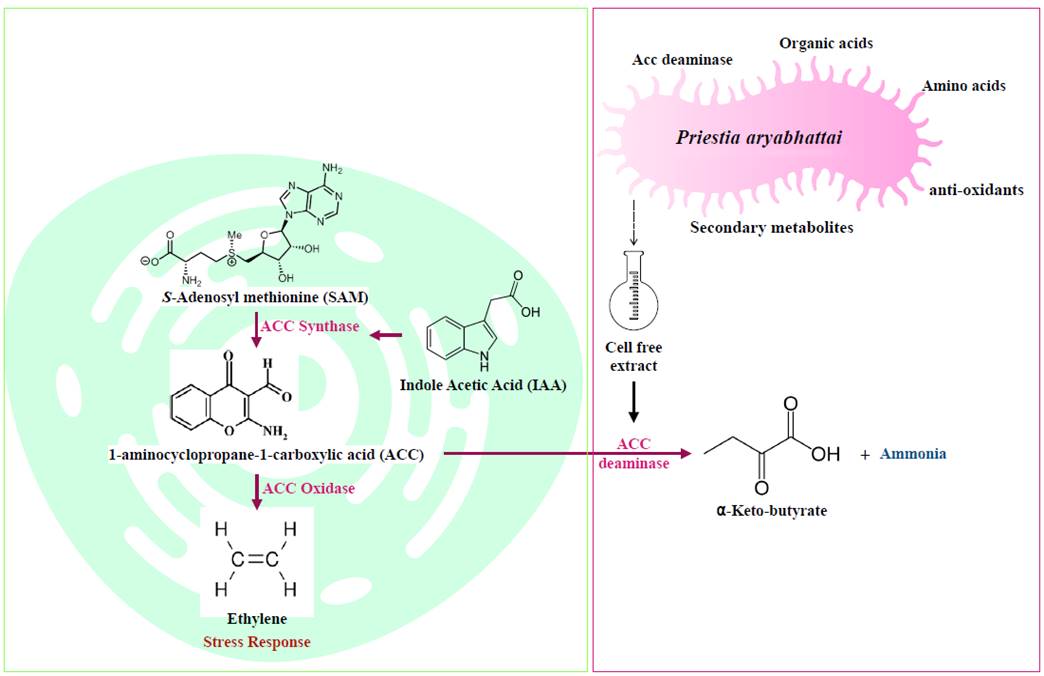

Supplement: Supplementary file 1 [file plants-15-01130-s001.zip › Supplementary Figure S1.jpg]
